# Supplementary material for: Magnetoelectric effect in organic molecular solids
Source: Sci Rep. 2016 Feb 15;6:20781. doi: 10.1038/srep20781 (PMC4753515; doi:10.1038/srep20781)
Supplement: Supplementary Information [file srep20781-s1.pdf]

Supplemental Information of
"Magnetoelectric effect in organic molecular solids"

Makoto Naka and Sumio Ishihara*

Department of Physics, Tohoku University, Sendai 980-8578, Japan.

**e-mail: ishihara@cmpt.phys.tohoku.ac.jp*

(Dated: December 14, 2015)

I. MODEL HAMILTONIAN

The model Hamiltonian for the molecular dimer systems introduced in equation (1) in the main text is derived from the extended Hubbard-type Hamiltonian[1,2]. A molecular dimer unit consisting of the two molecules termed a and b is introduced at each site in a crystal lattice. The Hamiltonian is given by $\mathcal{H}_{\rm EH} = \mathcal{H}_{\rm intra} + \mathcal{H}_{\rm inter}$. The intra-dimer term is given by

$$\mathcal{H}_{\rm intra} = t_A \sum_{i\sigma} \left(c^\dagger_{ia\sigma} c_{ib\sigma} + {\rm H.c.} \right) + U \sum_{i\mu} n_{i\mu\uparrow} n_{i\mu\downarrow} + V_A \sum_i n_{ia} n_{ib}, \tag{1}$$

in which $c^\dagger_{i\mu\sigma}$ ($c_{i\mu\sigma}$) is the creation (annihilation) operator for a hole with spin $\sigma(=\uparrow,\downarrow)$ and molecule $\mu(=a,b)$ at i-th dimer, and $n_{i\mu}(=\sum_\sigma n_{i\mu\sigma} = \sum_\sigma c^\dagger_{i\mu\sigma} c_{i\mu\sigma})$ is the number operator. We introduce the intra-dimer hopping integral (t_A), the electron-electron interaction inside a molecule (U), and the inter-molecule electron-electron interaction inside a dimer unit (V_A). The inter-dimer term is given by

$$\begin{aligned} \mathcal{H}_{\rm inter} &= \mathcal{H}_t + \mathcal{H}_V \\ &= \sum_{\langle ij \rangle \mu\mu'\sigma} t^{\mu\mu'}_{ij} \left(c^\dagger_{i\mu\sigma} c_{i\mu'\sigma} + {\rm H.c.} \right) + \sum_{\langle ij \rangle \mu\mu'} V^{\mu\mu'}_{ij} n_{i\mu} n_{j\mu'}, \end{aligned} \tag{2}$$

where $t^{\mu\mu'}_{ij}$ and $V^{\mu\mu'}_{ij}$, respectively, are the hopping integral and the electron-electron interaction between the molecule μ at i-th dimer unit and the molecule μ' at j-th dimer unit.

In the case of the strong electron-electron interaction inside the dimer, i.e. $U - t_A, V_A - t_A \gg t_{\mu\mu'}$, $V_{\mu\mu'}$, the number of hole at each dimer is fixed to be one, and the low-energy effective Hamiltonian is obtained by the perturbational calculations, in which $\mathcal{H}_{\rm inter}$ is treated as the perturbational term. We introduce the pseudo spin (PS) operator $\boldsymbol{Q}$ with an amplitude 1/2 to describe the charge degree of freedom inside the dimer defined by

$$\boldsymbol{Q}_i = \frac{1}{2} \sum_{\sigma\nu\nu'} \hat{c}^\dagger_{i\nu\sigma} \boldsymbol{\sigma}_{\nu\nu'} \hat{c}_{i\nu\sigma}, \tag{3}$$

where we define the hole operators for the bonding and antibonding molecular orbitals as $\hat{c}_{i\alpha(\beta)\sigma} = (c_{ia\sigma} - (+) c_{ib\sigma})/\sqrt{2}$. The eigen functions of Q^x are the charge polarized state where the a or b orbital is occupied by a hole, and those for Q^z are the non-polarized states where the antibonding orbital (α) or the bonding orbital (β) is occupied. The effective Hamiltonian up to the order of $\mathcal{O}(\mathcal{H}_V)$ and $\mathcal{O}(\mathcal{H}_t^2)$ is given by

$$\mathcal{H}_{\rm eff} = \widetilde{\mathcal{H}}_{\rm intra} + \widetilde{\mathcal{H}}_V + \mathcal{H}_J. \tag{4}$$

The first two terms are represented by

$$\widetilde{\mathcal{H}}_{\rm intra} + \widetilde{\mathcal{H}}_V = \Gamma \sum_i Q_i^z + \sum_{\langle ij \rangle} W_{ij} Q_i^x Q_j^x, \tag{5}$$

where $\Gamma(= 2t_A)$ is the intra-dimer electron transfer and $W_{ij}(= V_{ij}^{aa} + V_{ij}^{bb} - V_{ij}^{ab} - V_{ij}^{ba})$ is the Coulomb interaction. The third term in equation (4) represents the exchange interaction originating from the second order perturbation with respect to $\mathcal{H}_t$, and is classified by the spin-singlet and spin-triplet intermediate states in the perturbational processes as

$$\mathcal{H}_J = -\sum_{\langle ij \rangle} \left(\frac{3}{4} + \boldsymbol{S}_i \cdot \boldsymbol{S}_j \right) h_{ij}^T - \sum_{\langle ij \rangle} \left(\frac{1}{4} - \boldsymbol{S}_i \cdot \boldsymbol{S}_j \right) h_{ij}^S. \tag{6}$$

Explicit forms of h_{ij}^m $(m = T, S)$ are represented by

$$\begin{aligned} h_{ij}^m = & \sum_{\nu_1, \nu_2 = (\alpha, \beta)} J_{mij}^{\nu_1 \nu_2} \hat{n}_{i\nu_1} \hat{n}_{j\nu_2} + \sum_{\gamma_1, \gamma_2 = (+,-)} J_{mij}^{\gamma_1 \gamma_2} Q_i^{\gamma_1} Q_j^{\gamma_2} \\ & + \sum_{\nu = (\alpha, \beta)} \left(J_{mij}^{x\nu} Q_i^x \hat{n}_{j\nu} + J_{mij}^{\nu x} \hat{n}_{i\nu} Q_j^x \right), \end{aligned} \tag{7}$$

where $Q_i^{\pm} = Q_i^x \pm i Q_i^y$ and $\hat{n}_{i\nu} = \sum_{\sigma = (\uparrow, \downarrow)} \hat{c}_{i\nu\sigma}^\dagger \hat{c}_{i\nu\sigma}$. The exchange constants are given by $J_{Tij}^{\nu\nu} = (\hat{t}_{\alpha\beta}^2 + \hat{t}_{\beta\alpha}^2)/\Delta_{\nu\nu}^T$, $J_{Tij}^{\nu\bar{\nu}} = (\hat{t}_{\alpha\alpha}^2 + \hat{t}_{\beta\beta}^2)/\Delta_{\nu\bar{\nu}}^T$, $J_{Tij}^{++} = J_{Tij}^{--} = -\hat{t}_{\alpha\beta}\hat{t}_{\beta\alpha} \left(\Delta_{\alpha\alpha}^{S-1} + \Delta_{\beta\beta}^{S-1} \right)$, $J_{Tij}^{+-} = J_{Tij}^{-+} = -2\hat{t}_{\alpha\alpha}\hat{t}_{\beta\beta}/\Delta_{\alpha\beta}^T$, $J_{Tij}^{x\nu} = (\hat{t}_{\beta\beta}\hat{t}_{\alpha\beta} - \hat{t}_{\alpha\alpha}\hat{t}_{\beta\alpha}) \left(\Delta_{\nu\nu}^{T-1} + \Delta_{\alpha\beta}^{T-1} \right)$, and $J_{Tij}^{\nu x} = J_{Tij}^{x\nu} \left(\hat{t}_{\nu\nu'} \leftrightarrow \hat{t}_{\nu'\nu} \right)$ for the spin-triplet intermediate states, and

$$J_{Sij}^{\nu\nu} = \frac{\hat{t}_{\alpha\beta}^2 + \hat{t}_{\beta\alpha}^2}{\Delta_{\nu\nu}^S} + 4\hat{t}_{\nu\nu}^2 \left(\frac{D_\nu^2}{\Delta_{\nu\nu}^{D+}} + \frac{D_{\bar{\nu}}^2}{\Delta_{\nu\nu}^{D-}} \right), \tag{8}$$

$$J_{Sij}^{\nu\bar{\nu}} = \frac{\hat{t}_{\alpha\alpha}^2 + \hat{t}_{\beta\beta}^2}{\Delta_{\alpha\beta}^S} + 2\hat{t}_{\nu\bar{\nu}}^2 \left(\frac{1}{\Delta_{\alpha\beta}^{D+}} + \frac{1}{\Delta_{\alpha\beta}^{D-}} \right), \tag{9}$$

$$J_{Sij}^{++} = J_{Sij}^{--} = \hat{t}_{\alpha\beta}\hat{t}_{\beta\alpha} \left(\frac{1}{\Delta_{\alpha\alpha}^S} + \frac{1}{\Delta_{\beta\beta}^S} \right) + 2\hat{t}_{\alpha\alpha}\hat{t}_{\beta\beta} C_+ C_- \left(\frac{1}{\Delta_{\alpha\alpha}^{D+}} + \frac{1}{\Delta_{\beta\beta}^{D+}} - \frac{1}{\Delta_{\alpha\alpha}^{D-}} - \frac{1}{\Delta_{\beta\beta}^{D-}} \right), \tag{10}$$

$$J_{Sij}^{+-} = J_{Sij}^{-+} = \frac{2\hat{t}_{\alpha\alpha}\hat{t}_{\beta\beta}}{\Delta_{\alpha\beta}^S} + 4\hat{t}_{\alpha\beta}\hat{t}_{\beta\alpha} C_+ C_- \left(\frac{1}{\Delta_{\alpha\beta}^{D+}} - \frac{1}{\Delta_{\alpha\beta}^{D-}} \right), \tag{11}$$

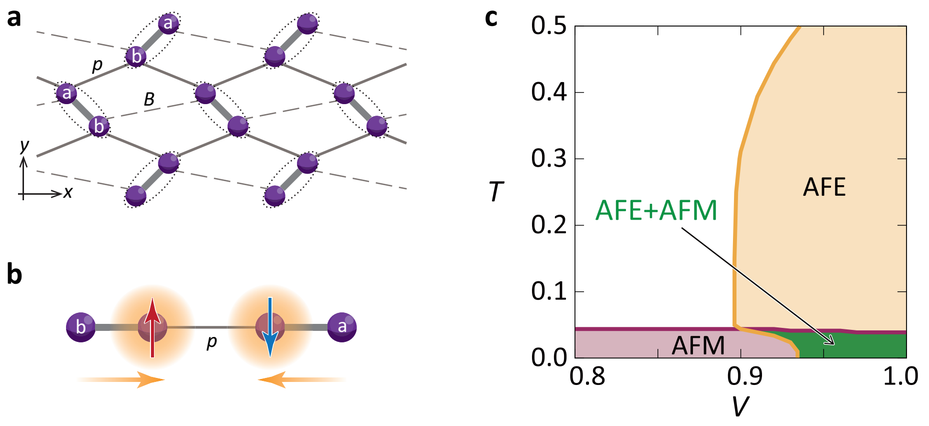

FIG. 1: **Lattice structure of the κ-type BEDT-TTF compounds and the dominant exchange interaction.** ***a***, A schematic lattice structure where filled circles denoted by a and b represent the BEDT-TTF molecules. Symbols B and p are the names of the bonds. Dotted ovals denote the dimer units. ***b***, A stable spin and charge configuration in the nearest neighboring two dimer units connected by the p bond. Filled circles, shaded circles, thin arrows, and shaded arrows indicate molecules, charge distributions, spins, and electric dipoles, respectively. ***c***, A phase diagram calculated based on the Hamiltonian in equation (4), where all of the exchange interaction terms are taken into account.

$$J_{Sij}^{x\nu} = (\hat{t}_{\beta\beta}\hat{t}_{\alpha\beta} + \hat{t}_{\alpha\alpha}\hat{t}_{\beta\alpha}) \left(\frac{1}{\Delta_{\nu\nu}^{S}} + \frac{1}{\Delta_{\alpha\beta}^{S}} \right) + 2\hat{t}_{\nu\nu}\hat{t}_{\bar{\nu}\nu} \left\{ D_\nu (D_\nu + D_{\bar{\nu}}) \left(\frac{1}{\Delta_{\nu\nu}^{D+}} + \frac{1}{\Delta_{\alpha\beta}^{D+}} \right) - D_\nu (D_\nu - D_{\bar{\nu}}) \left(\frac{1}{\Delta_{\nu\nu}^{D-}} + \frac{1}{\Delta_{\alpha\beta}^{D-}} \right) \right\}, \quad (12)$$

and $J_{Sij}^{\nu x} = J_{Sij}^{x\nu} \left(\hat{t}_{\nu\nu'} \leftrightarrow \hat{t}_{\nu'\nu} \right)$ for the spin-singlet intermediate states. We define $\bar{\nu} = (\beta, \alpha)$ for $\nu = (\alpha, \beta)$, and $\hat{t}_{\nu\nu'} \equiv \hat{t}_{ij}^{\nu\nu'} = \sum_{\mu,\mu'=(a,b)} U_{\nu\mu} t_{ij}^{\mu\mu'} U_{\mu'\nu'}^{\dagger}$. Energy differences are defined by $\Delta_{\nu\nu'}^{T} = E_T - E_\nu - E_{\nu'}$, $\Delta_{\nu\nu'}^{S} = E_S - E_\nu - E_{\nu'}$, and $\Delta_{\nu\nu'}^{D\pm} = E_{D\pm} - E_\nu - E_{\nu'}$ with $E_T = V_A$, $E_S = U$, $E_{D\pm} = (U + V_A)/2 \pm \sqrt{4t_A^2 + (U - V_A)^2/4}$, $E_\alpha = -t_A$, and $E_\beta = t_A$. Coefficients are $D_{\nu(\bar{\nu})} = \left\{ C_{+(-)}, C_{-(+)} \right\}$ for $\nu = (\alpha, \beta)$, where $C_+^2 + C_-^2 = 1$ and $C_-/C_+ = \left\{ 2E_{D+} + 4t_A - (U + V_A) \right\} / (U - V_A)$ are satisfied.

We apply this model to a two-dimensional plane in the crystal lattice of the κ-type BEDT-

TTF compounds shown in Fig. 1$\boldsymbol{a}$. We focus on the ME effect in the AFE+AFM ordered phase. The calculated results are shown in Fig. 2 in the main text. Analyses of the ME effect without the conventional magnetic long-range orders will be discussed in Sect. III, and the results are shown in Fig. 4 in the main text. In the numerical calculations, parameter values are chosen to be $U = 6$ and $V_A = 4.5$, where t_A is taken as a unit of energy. As for the inter-dimer hopping integral, the most dominant term on a bond denoted by p shown in Fig. 1$\boldsymbol{a}$ is taken into account. A numerical value of the hopping integral is chosen to be $t_p = 0.5$. This simplification might correspond to the κ-type BEDT-TTF compounds showing the AFM order, for example κ-(BEDT-TTF)$_2$Cu[N(CN)$_2$]Cl, where the so-called magnetic frustration effect is weak[3].

The dominant exchange interactions in addition to the unperturbed terms in equation (5) are given by

$$\mathcal{H} = \Gamma \sum_i Q_i^z - V \sum_{\langle ij \rangle} Q_i^x Q_j^x + J \sum_{\langle ij \rangle} \boldsymbol{S}_i \cdot \boldsymbol{S}_j - K \sum_{\langle ij \rangle} \boldsymbol{S}_i \cdot \boldsymbol{S}_j Q_i^x Q_j^x, \tag{13}$$

which is the Hamiltonian introduced in equation (1) in the main text. The third and fourth terms describe the Heisenberg-type exchange interaction and the spin-charge coupling, respectively. The exchange constants are explicitly given by

$$J = \frac{1}{4} \sum_{\nu_1, \nu_2 = (\alpha, \beta)} \left(J_{Sij}^{\nu_1 \nu_2} - J_{Tij}^{\nu_1 \nu_2} \right) \tag{14}$$

and

$$K = \sum_{\gamma_1, \gamma_2 = (+,-)} \left(J_{Sij}^{\gamma_1 \gamma_2} - J_{Tij}^{\gamma_1 \gamma_2} \right). \tag{15}$$

The third and fourth terms in equation (13) in an isolated p bond favors the antiparallel alignments of spins and charge PSs, as shown in Fig. 1$\boldsymbol{b}$. This originates from the kinetic energy gain of t_p. Amplitudes of J and K are about 2–10 times larger than other exchange constants. The phase diagram where all exchange interaction terms are taken into account is shown in Fig. 1$\boldsymbol{c}$. The phase diagram calculated based on the Hamiltonian in equation (13) shown in Fig. 2$\boldsymbol{a}$ in the main text well reproduces the result in Fig. 1$\boldsymbol{c}$, implying the relevance of the present Hamiltonian in equation (13).

II. MEAN-FIELD APPROXIMATION

The Hamiltonian introduced in equation (1) in the main text is analyzed by the mean-field (MF) approximation. We take the unit cell that includes the two nonequivalent dimers, and introduce the following MFs, $\langle S^\mu \rangle$, $\langle Q^x \rangle$, and $\langle S^\mu Q^x \rangle$ with $\mu = (x, y, z)$ in each dimer, where the bracket represents the thermal average. The interaction terms are decoupled as $\boldsymbol{S}_i \cdot \boldsymbol{S}_j \to \boldsymbol{S}_i \cdot \langle \boldsymbol{S}_j \rangle + \langle \boldsymbol{S}_i \rangle \cdot \boldsymbol{S}_j - \langle \boldsymbol{S}_i \rangle \cdot \langle \boldsymbol{S}_j \rangle$, $Q_i^x Q_j^x \to Q_i^x \langle Q_j^x \rangle + \langle Q_i^x \rangle Q_j^x - \langle Q_i^x \rangle \langle Q_j^x \rangle$, and $\boldsymbol{S}_i Q_i^x \cdot \boldsymbol{S}_j Q_j^x \to \boldsymbol{S}_i Q_i^x \cdot \langle \boldsymbol{S}_j Q_j^x \rangle + \langle \boldsymbol{S}_i Q_i^x \rangle \cdot \boldsymbol{S}_j Q_j^x - \langle \boldsymbol{S}_i Q_i^x \rangle \cdot \langle \boldsymbol{S}_j Q_j^x \rangle$. The MFs are determined selfconsistently.

III. RANDOMNESS AND CLUSTER MEAN-FIELD APPROXIMATION

Randomness is introduced as the random electric field, which couples to the dimer dipoles. This interaction is represented by

$$\mathcal{H}_{\mathrm{r}} = -\sum_i h_i Q_i^x, \tag{16}$$

where h_i is the random electric field at the i-th dimer and is defined as $h_i = h\epsilon_i$ with amplitude h and the site-depend random variable $\epsilon_i = \pm 1$. This is determined by the bimodal distribution function given by

$$P(\epsilon_i) = \frac{1}{2}\left\{\delta(\epsilon_i - 1) + \delta(\epsilon_i + 1)\right\}. \tag{17}$$

An expectation value of an observable $\mathcal{A}$ is given by the configuration average defined by

$$[\langle \mathcal{A} \rangle] = \prod_i \int_{-\infty}^{\infty} dh_i P(\epsilon_i) \langle \mathcal{A} \rangle (\{h_i\}), \tag{18}$$

where $\langle \mathcal{A} \rangle (\{h_i\})$ is the thermal average for a certain random field configuration $\{h_i\}$[4].

A sum of the effective Hamiltonian introduced in equation (13) in Sect. I and the random field term in equation (16) is adopted as the model Hamiltonian. We examine a possibility of the ME effects without conventional magnetic long-range orders, in contrast to the ME effect in the AFM ordered phase shown in Fig. 2 in the main text. Thus, we introduce the magnetic frustration effect, in addition to the randomness effects, and suppress development of the Néel order. Then, the exchange constants in equation (13) are estimated by considering the hopping integrals for the B bonds, as well as those in the p bonds (see Fig. 1***a***). Numerical values of the hopping integrals are chosen to be $t_B = t_p = 0.5$. This might be suitable for the κ-type BEDT-TTF compounds, in which no conventional magnetic long-range order appears, for example κ-(BEDT-TTF)$_2$Cu$_2$(CN)$_3$, where the frustration effect is strong[3].

The Hamiltonian with the random electric field is analyzed by the cluster MF approximation, in which the exact diagonalization methods based on the Householder algorithm and the MF approximation are combined. The MF decouplings are introduced as $\boldsymbol{S}_i \cdot \boldsymbol{S}_j \to \boldsymbol{S}_i \cdot [\langle \boldsymbol{S}_j \rangle] + [\langle \boldsymbol{S}_i \rangle] \cdot \boldsymbol{S}_j - [\langle \boldsymbol{S}_i \rangle] \cdot [\langle \boldsymbol{S}_j \rangle]$, $Q_i^x Q_j^x \to Q_i^x \left[\langle Q_j^x \rangle\right] + \left[\langle Q_i^x \rangle\right] Q_j^x - \left[\langle Q_i^x \rangle\right] \left[\langle Q_j^x \rangle\right]$, and $\boldsymbol{S}_i Q_i^x \cdot \boldsymbol{S}_j Q_j^x \to \boldsymbol{S}_i Q_i^x \cdot \left[\langle \boldsymbol{S}_j Q_j^x \rangle\right] + \left[\langle \boldsymbol{S}_i Q_i^x \rangle\right] \cdot \boldsymbol{S}_j Q_j^x - \left[\langle \boldsymbol{S}_i Q_i^x \rangle\right] \cdot \left[\langle \boldsymbol{S}_j Q_j^x \rangle\right]$. A cluster including three dimer units with the periodic boundary condition is adopted. The spin-glass and charge-glass order parameters plotted in Fig. 4***b*** in the main text are defined as

$$q_S = \sqrt{[\langle S^x \rangle^2] + [\langle S^y \rangle^2] + [\langle S^z \rangle^2]} \tag{19}$$

and

$$q_Q = \sqrt{[\langle Q^x \rangle^2]}, \tag{20}$$

respectively.

[1] Naka, M. and Ishihara, S. Electronic ferroelectricity in a dimer Mott insulator. *J. Phys. Soc. Jpn.* **79,** 063707 (2010).

[2] Hotta, C. Quantum electric dipoles in spin-liquid dimer Mott insulator κ-(ET)$_2$Cu$_2$(CN)$_3$. *Phys. Rev. B* **82,** (R)241104 (2010).

[3] Koretsune, T. and Hotta, C. Evaluating model parameters of the κ- and β'-type Mott insulating organic solids. *Phys. Rev. B* **89,** 045102 (2014).

[4] Nishimori, H. and Oritiz, G. *Elements of Phase Transitions and Critical Phenomena* (Oxford University Press, Oxford, 2011).
